# 1001. Case Study. Managing Pain in Pediatric Patients Using a Bioresorbable Lidocaine-Silver Matrix: A Case Series

**DOI:** 10.1093/jbcr/irag033.189

**Published:** 2026-04-06

**Authors:** Katrina L Weaver

**Affiliations:** Logan Health Children's, Kalispell, Montana

## Abstract

**Patient Presentation (age range, injury details, relevant history):**

Two pediatric patients treated at a regional referral center were evaluated: one for a partial-thickness burn and one for an autograft donor site. Patient 1 was a 16-year-old female with a 3% TBSA partial-thickness scald injury on her dorsal foot. Patient 2 was a 5-year-old male who suffered a 4% TBSA mixed-depth contact burn.

**Clinical Challenges:**

Effective pain control in pediatric burn patients is critical for both immediate comfort and long-term recovery, yet management is complicated by communication barriers, patient anxiety, and risks of opioid-related side effects. To address these challenges, a conformable, bioresorbable polymer matrix combining lidocaine HCl for initial analgesia with metallic/ionic silver to reduce infection risk was developed. This case series examines preliminary effectiveness in pediatric wound management and pain control.

**Management Approach:**

The bioresorbable matrix was applied in lieu of bupivacaine injection. For patient 1, the burn wound was debrided, and the matrix was applied beneath a temporary skin substitute, followed by absorbent dressing, gauze bandage, and self-adherent wrap. For patient 2, the donor site wound was sprayed with skin cell suspension autograft prior to application of the matrix, followed by antibiotic ointment-coated petrolatum gauze, absorbent dressing, gauze bandage, and self-adherent wrap. Pain, infection, and healing outcomes were documented.

**Outcomes:**

In the 24 hours prior to debridement and matrix application, patient 1 reported pain scores of nine or greater and was unable to sleep, requiring narcotics for pain relief. Following the operation, pain medications were limited to acetaminophen and NSAIDs. She reported pain as substantially better than the night prior, with her pain score reducing to 2 within 3-5 hours, and she was able to attend social events the same day. Patient 2, following application of the matrix to his donor site wound on his posterior thigh/buttocks, he reported no pain at the donor site at one-hour post-operation and throughout his hospital stay. No infection developed in either patient; ^2^wounds were healed at post-operative day 10.

**Lessons Learned:**

This two-patient case series demonstrates feasibility of a bioresorbable antimicrobial matrix with lidocaine HCl in pediatric burn care to support pain control, eliminating the need for narcotics and bupivacaine injection. Benefits extended beyond initial comfort to enable same-day return to activities, suggesting potential to reduce long-term pain burden. Future research should assess efficacy in a randomized controlled trial.

**Applicability to Practice:**

If validated in larger trials, this bioresorbable matrix may simplify pain management, reduce opioid exposure, and optimize recovery in pediatric burn patients.

